# Supplementary material for: Identification, Culture Characteristics and Whole-Genome Analysis of Pestalotiopsis neglecta Causing Black Spot Blight of Pinus sylvestris var. mongolica
Source: J Fungi (Basel). 2023 May 12;9(5):564. doi: 10.3390/jof9050564 (PMC10219049; doi:10.3390/jof9050564)
Supplement: Supplementary file 1 [file jof-09-00564-s001.zip › jof-2337088-supplementary.pdf]

Table S1. Sequence identity statistics of the *P. neglecta* strain YJ-3 genome annotated in NCBI nr with identify of 100%.

Gene ID	Location	Hit[1]	Hit-Description	Identity
gene00071	Scaffold1	XP_007841584.1	40S ribosomal protein S26E	100
gene00185	Scaffold1	XP_007839240.1	Mitochondrial import inner membrane translocase subunit TIM9	100
gene00632	Scaffold1	XP_007841410.1	Ubiquitin-conjugating enzyme	100
gene00716	Scaffold1	KAF3015326.1	ribosomal protein S14, S11	100
gene00832	Scaffold1	XP_007839759.1	Guanine nucleotide-binding protein subunit alpha	100
gene01159	Scaffold1	KAF3007103.1	GTP-binding protein	100
gene01291	Scaffold1	XP_007832045.1	60S ribosomal protein L29	100
gene02147	Scaffold1	WP_106631623.1	30S ribosomal protein S19	100
gene02304	Scaffold1	XP_007828274.1	ADP-ribosylation factor	100
gene02619	Scaffold2	XP_007832746.1	ADP-ribosylation factor 6	100
gene03161	Scaffold2	XP_007832969.1	DNA-directed RNA polymerases I, II, and III subunit RPABC5	100
gene03577	Scaffold2	XP_007833905.1	ribonucleoprotein-associated protein	100
gene04167	Scaffold2	XP_003712412.1	60S ribosomal protein L23	100
gene04243	Scaffold2	KAF3014175.1	Serine/threonine-protein kinase smg1	100
gene04590	Scaffold2	XP_007840519.1	ER-derived vesicles protein ERV14	100
gene05125	Scaffold3	KAF3007209.1	Vacuolar protein sorting-associated protein 26	100
gene05818	Scaffold3	XP_007832302.1	40S ribosomal protein S22	100
gene06158	Scaffold3	KAF3024254.1	DNA-directed RNA polymerase I core subunit rpa12	100
gene06205	Scaffold3	XP_007831955.1	proteasome subunit alpha type-5	100
gene06935	Scaffold4	XP_007835576.1	40S ribosomal protein S5	100
gene06984	Scaffold4	XP_007835667.1	hypothetical protein	100
gene06985	Scaffold4	XP_007835668.1	Cytochrome c oxidase subunit 6B	100
gene07556	Scaffold4	XP_007835832.1	40S ribosomal protein S23	100
gene07580	Scaffold4	KAF3025461.1	Cytosolic copper metallochaperone	100
gene07910	Scaffold4	XP_018152220.1	histone H3	100
gene07910	Scaffold4	WP_139128006.1	histone H3	100
gene07910	Scaffold4	KKP01815.1	histone H3	100
gene07910	Scaffold4	TDZ22988.1	histone H3	100
gene07910	Scaffold4	WP_069696828.1	MULTISPECIES: histone H3	100
gene07911	Scaffold4	CRK42577.1	hypothetical protein	100
gene07911	Scaffold4	XP_018152221.1	Histone H4	100
gene07911	Scaffold4	XP_001548661.1	hypothetical protein	100
gene07941	Scaffold4	KAF3025989.1	nucleoside diphosphate kinase	100
gene07941	Scaffold4	WP_106474479.1	nucleoside-diphosphate kinase	100
gene07941	Scaffold4	XP_007836394.1	hypothetical protein	100
gene08068	Scaffold4	XP_007837893.1	Small nuclear ribonucleoprotein Sm D3	100
gene08656	Scaffold5	XP_007828906.1	Trafficking protein particle complex subunit BET3	100
gene08869	Scaffold5	XP_007829285.1	40S ribosomal protein S21	100
gene09150	Scaffold5	XP_028463238.1	hypothetical protein	100

[1] Name of target sequence matched in NR library.

Table S2. CYP51 genes in *P. neglecta* strain YJ-3 genome.

Gene ID	Location	Superfamily	Homologous Family	Identity(%)
gene00818	Scaffold1	CYP51	CYP51-like	26.6
gene01030	Scaffold1	CYP51	CYP51-like	26.9
gene01475	Scaffold1	CYP51	CYP51-like	36
gene01506	Scaffold1	CYP51	CYP51-like	30.9
gene01777	Scaffold1	CYP51	CYP51-like	32.9
gene02155	Scaffold1	CYP51	CYP51-like	58.9
gene02309	Scaffold1	CYP51	CYP51-like	32.6
gene03679	Scaffold2	CYP51	CYP51-like	39.8
gene04311	Scaffold2	CYP51	CYP51-like	22.9
gene04398	Scaffold2	CYP51	CYP51-like	30.7
gene04445	Scaffold2	CYP51	CYP51-like	29.4
gene04449	Scaffold2	CYP51	CYP51-like	35
gene04832	Scaffold3	CYP51	CYP51-like	29.5
gene05010	Scaffold3	CYP51	CYP51-like	33
gene05089	Scaffold3	CYP51	CYP51-like	31.7
gene05771	Scaffold3	CYP51	CYP51-like	93.7
gene05793	Scaffold3	CYP51	CYP51-like	32.9
gene06073	Scaffold3	CYP51	CYP51-like	37.3
gene06187	Scaffold3	CYP51	CYP51-like	30.9
gene07238	Scaffold4	CYP51	CYP51-like	33.8
gene07505	Scaffold4	CYP51	CYP51-like	30.7
gene08521	Scaffold4	CYP51	CYP51-like	27.1
gene08596	Scaffold4	CYP51	CYP51-like	31.4
gene08912	Scaffold5	CYP51	CYP51-like	39.2
gene09152	Scaffold5	CYP51	CYP51A-like	31.8
gene09208	Scaffold5	CYP51	CYP51-like	36
gene09240	Scaffold5	CYP51	CYP51-like	35.2
gene09381	Scaffold5	CYP51	CYP51-like	29.2
gene09593	Scaffold5	CYP51	CYP51-like	33.2
gene10114	Scaffold5	CYP51	CYP51-like	32.7
gene10221	Scaffold5	CYP51	CYP51-like	32.3
gene10327	Scaffold5	CYP51	CYP51-like	28.6
gene10729	Scaffold6	CYP51	CYP51-like	36.8
gene11014	Scaffold6	CYP51	CYP51-like	28.3
gene11128	Scaffold6	CYP51	CYP51-like	38.4
gene11674	Scaffold6	CYP51	CYP51-like	32.6
gene11849	Scaffold6	CYP51	CYP51-like	29.8
gene11942	Scaffold6	CYP51	CYP51-like	32.2
gene12596	Scaffold7	CYP51	CYP51-like	27.1
gene13042	Scaffold7	CYP51	CYP51-like	82.8
gene13282	Scaffold7	CYP51	CYP51-like	31.6
gene13514	Scaffold7	CYP51	CYP51-like	33
gene13782	Scaffold7	CYP51	CYP51-like	30.7
gene13820	Scaffold7	CYP51	CYP51A-like	26.1
gene13866	Scaffold7	CYP51	CYP51-like	32.4
gene13911	Scaffold7	CYP51	CYP51-like	29.1
